# Supplementary material for: Association between atherogenic index of plasma and depression in premenopausal and postmenopausal women: A cross-sectional study
Source: PLoS One. 2025 Jul 2;20(7):e0327103. doi: 10.1371/journal.pone.0327103 (PMC12221011; doi:10.1371/journal.pone.0327103)
Supplement: Attachment — Submitted filename: Response to Reviewers.docx [file pone.0327103.s002.docx]

Dear Editor and Reviewer,

On behalf of all the contributing authors, I would like to express our sincere appreciation of your constructive comments concerning our article entitled *Association between Atherogenic Index of Plasma and depression in premenopausal and postmenopausal women: A cross-sectional study* (Manuscript ID: PONE-D-25-15440). We have carefully revised the manuscript to enhance its clarity and facilitate the understanding of the readers. Our point-to-point responses are presented in the following. We hope that the revision will satisfactorily address the comments and concerns of the editors and reviewers.

Our manuscript had been revised to meet PLOS ONE's stylistic requirements. According to the requirements of the journal, we remove any funding-related text from the manuscript and include a funding statement in the cover letter. Changes to our manuscript were all highlighted within the document by using yellow padding.

Editor#

General comments

Question: Authors should however improve on the discussion part finding plausible explanations to support the results obtained or otherwise. There are few situations in text that need to be supported by citation/references.

Response: Thanks for the comment. We had added discussion sections and added reference citations. We interpreted the finding that the risk of depression in postmenopausal women was reduced when the AIP was > 0.60.

Abstract

Question: There are some few studies that has explored AIP and depression in postmenopause (see PMID: 39648880 ....... there are few others). Avoid these emphatic claims. I suggest we keep the second sentence (aim) only.

Response: Thanks for the comments and we agree with you. We keep the second sentence only.

Introduction

Question: Estrogen deficiency in m ADDIN EN.CITE on in hepatocytes, .........There appears something missing here. Authors should 'fix' this sentence.

Response: Thanks. We had amended the sentence ‘Estrogen deficiency in menopausal women decreases estrogen receptor α expression in hepatocytes, resulting in altered expression of key enzymes and genes involved in lipid synthesis and catabolism, and increased serum levels of total cholesterol, triglycerides (TG), and low-density lipoprotein (LDL), and decreased levels of high-density lipoprotein (HDL)’.

Discussion

Question 1: Could we have some suggested explanations or discussion on why at AIP> 0.60, we have reduced risk of depression among the postmenopausal cohort. Is it due to chance (I doubt)?, the characteristics of the study cohort? This is pivotal and needs to be discussed.

Response 1: Thanks for the valuable comments. We reviewed the literature on postmenopausal women's susceptibility to depression and the association of AIP and depression, and added relevant content and references where possible. According to the estrogen withdrawal theory, the likely reason for this is that estrogen levels in postmenopausal women decline significantly and fluctuate almost imperceptibly, and their effect on depression is stronger than the effect of atherosclerosis on depression. Another possible reason lies in the limitations of the study sample. In the population of postmenopausal women, the sample size of AIP > 0.6 was only 67, and the large difference in sample size has a non-negligible impact on the results. Due to the specificity of the postmenopausal period, the relationship between AIP and depression in the postmenopausal female population seems to be more complex compared to premenopausal women. In future studies, we should incorporate both estrogens as well as indicators of atherosclerosis to further explore the mechanisms of depression in postmenopausal women.

Question 2: Authors should revisit the limitation section. Comment on the unavailability of data on estrogen and other endocrine and biomarkers and how they would have impacted findings.....

Response 2: Thanks. Because complete years of estrogen data are not available in the NHANES database, we were unable to verify the relationship between estrogen levels and AIP at different menopausal states and whether estrogen levels affect the relationship between AIP and depression.

Reviewer #

Introduction

Question: I would suggest to add relevant reference for increased susceptibility of postmenopausal women to Depression and also Association of AIP and depression among women in general.if any.

Response: Thanks. We reviewed the literature on postmenopausal women's susceptibility to depression and added relevant content and references as possible.

Methods

Question: The location of study may be mentioned in the methods section.

Also elaborate on how the samples are collected.

There is lack of information regarding the potential confounders and how they are handled ( describe the models).

Response: Thanks. We had added the location of study as well as detailed instructions on how to collect samples. We handled how to deal with potential confounders.

Results

Question: Overweight and Obesity are known risk of metabolic syndrome and also atherosclerosis. Would like to know the influence of weight/BMI on the results.

Response: Thanks for the valuable comments. We divided into two groups according to BMI (BMI < 24 kg/m2 and BMI ≥24 kg/m2) and conducted subgroup analyses. We did not find an interaction in premenopausal and postmenopausal women.

Limitations

Question: Though mentioned, would suggest to list and discuss all the limitations of the study including the confounders, information bias, self reporting bias etc.

Response: Thanks for the comments. We list the limitations of the study as much as possible.

Thank you once again for your valuable feedback. I look forward to receiving further comments from you. If you have any further questions or need further discussion, please feel free to contact me at any time.

Yours sincerely,

All authors
